# Chemotherapy use near the end-of-life in patients with metastatic breast cancer

**DOI:** 10.1007/s10549-020-05663-w

**Published:** 2020-05-07

**Authors:** Luisa Edman Kessler, Johnny Sigfridsson, Dora Hatzidaki, Jonas Bergh, Theodoros Foukakis, Vasilios Georgoulias, Alexios Matikas

**Affiliations:** 1 Department of Oncology/Pathology, Karolinska Institutet, Stockholm, Sweden; 2 Breast Cancer, Endocrine Tumours and Sarcoma Section, Theme Cancer, Karolinska University Hospital Solna, Eugeniavägen 3, 171 76 Stockholm, Sweden; 3 Hellenic Oncology Research Group, Athens, Greece

**Keywords:** Breast cancer, Chemotherapy, End-of-life, Futile, Metastatic

## Abstract

**Introduction:**

Very few data are available regarding the use of chemotherapy in patients with metastatic breast cancer (MBC) near the end-of-life, i.e., the final month. The aim of this study was to provide a descriptive analysis of its use in two different European geographic areas (Sweden and Greece).

**Materials and methods:**

We retrospectively collected data regarding clinicopathologic characteristics, survival, and use of chemotherapy during the final 30 days of life using two sources: for the Swedish cohort, patients who were diagnosed with MBC in 2010–2015 were identified from the Stockholm-Gotland population-based Breast Cancer Registry and treatment data were collected using hospital charts. For the Greek cohort, patients with MBC were identified from hospital charts at two hospitals in Athens and Crete.

**Results:**

In the Swedish cohort, 1571 patients were identified; median overall survival was 16.96 months (95% CI 15.4–18.4). 23.2% of patients were treated with chemotherapy during the final month of life, with higher rates among patients ≤ 60 years (*p* < 0.001). Per OS monotherapy such as capecitabine or vinorelbine was most commonly used. In contrast, median OS in the Greek cohort (*n* = 966) was 49.8 months (95% CI 45.6–54.1) and 46.5% of patients received chemotherapy at the end-of-life, most commonly intravenous drug combinations. In multivariable analysis, age and albumin levels were statistically significantly associated with chemotherapy use in the Swedish cohort.

**Conclusion:**

Chemotherapy use near the end-of-life was common, which might negatively impact patient quality of life.

**Electronic supplementary material:**

The online version of this article (10.1007/s10549-020-05663-w) contains supplementary material, which is available to authorized users.

## Implications for practice

Although various options are available for the treatment of patients with metastatic breast cancer, chemotherapy is commonly used in later stages of the disease. Very few data however inform on the patterns of chemotherapy use during the final month of life, which may negatively impact quality of life and cause unnecessary toxicity. With this retrospective descriptive analysis, we analyze the types of chemotherapy used during the final month of life and the associated patient clinical and demographic characteristics in two European countries, Sweden, and Greece. Being one of the few studies that have surveyed chemotherapy use in breast cancer patients at this stage of the disease, it provides important background for mitigation strategies.

## Introduction

Metastatic breast cancer (MBC) is with few exceptions an incurable disease, where treatment aims at prolonging survival and improving quality of life by relieving symptoms. Several treatment approaches are available for MBC, including endocrine manipulation, radiotherapy, and chemotherapy. Advances in the understanding of BC biology in recent decades and the deciphering of the plasticity of the clonal architecture of BC under the natural selection pressure applied by anticancer therapy [1–3] have led to the development and approval of several targeted agents, such as anti-HER2 (human epidermal growth factor receptor 2) therapy [4], mTOR- (mechanistic target of rapamycin) and CDK4/6 (cyclin-dependent kinase 4/6) inhibitors [5], as well as immunotherapy through checkpoint inhibition [6].

Despite these improvements, the vast majority of MBC patients will receive non-targeted cytotoxic therapy during the disease trajectory, most often in the form of continuous mono-chemotherapy until disease progression, at which point treatment is changed to a non-cross resistant agent. There is limited randomized evidence on the treatment of this typically heavily pretreated population, with eribulin being the only single agent demonstrating an overall survival (OS) benefit in patients that had received at least two chemotherapy lines for advanced disease [7]. It is also important to note that the heavily pretreated MBC population likely differs from that of patients enrolled in clinical trials, the latter generally being fit, younger and without significant comorbidities. This poses a difficulty in translating results from clinical trials to the general MBC population where the observed median OS is shorter [8], and clearly supports a need to gain knowledge about real-life use of chemotherapy in late-stage MBC. This lack of evidence and consistent clinical guidelines may expose patients to unnecessarily aggressive and ultimately futile treatment, which can negatively impact the patients’ quality of life [9].

To date, the use of chemotherapy in MBC patients near the end-of-life has not been extensively studied, and there are no well-described predictors for the administration of futile chemotherapy in this patient group. Furthermore, the lack of consistent guidelines for this population may result in treatment differences between cancer centers, with variations being possibly susceptible to cultural differences between different countries. In this study, we explore the real-life use of chemotherapy near the end-of-life and the characteristics of MBC patients treated at this stage in areas of Sweden (Stockholm) and Greece (Athens and Heraklion), in order to highlight possible factors that could help in decision-making and identify cultural differences between Northern and Southern Europe that may drive palliative care.

## Materials and methods

### Study design

This is a retrospective cohort study whose primary objective was to describe the contemporary use of futile chemotherapy among patients with MBC, defined in this study as chemotherapy administered within the last 30 days of the patient’s life. The secondary objectives of the study were to identify potential predictive factors for the use of any chemotherapy or the initiation of a new chemotherapy towards the end of the patient’s life; and to provide an update regarding the survival at the population level of patients diagnosed with MBC. Patients included in the study had been treated for MBC in the Stockholm-Gotland region, Sweden as well as in two hospitals in Greece: University Hospital of Heraklion, Crete and Metropolitan General Hospital, Athens. The study was approved by ethics committees in both Sweden (decision number 2016/1303/31 and 2018/642-32) and Greece (decision number 2019/151).

### Patients and data collection

#### Sweden

We identified patients aged 18 years older who received the diagnosis of MBC between January 1, 2010 and December 31, 2015 using the Stockholm-Gotland breast cancer registry (SBCR). The SBCR is a population-based registry, which contains demographic and clinicopathologic information regarding all newly diagnosed cases of BC in the region since 1976. It has an excellent completeness of 99% among patients aged 75 years or younger at the time of diagnosis. Data collected from the registry were date of metastatic disease diagnosis and clinicopathologic characteristics such as ER, PR and HER2 status from the primary tumor [10]. For this study, data were also manually collected from individual charts of the previously identified patients, accessed through the electronic hospital chart system Take-Care which is common for all hospitals in the Stockholm–Gotland region. Information was collected regarding date of death or of last follow-up, chemotherapy use near the end-of-life, type of chemotherapy regimen given and whether a new regimen was initiated. Albumin was used as a surrogate marker for performance status [11], and the plasma albumin value closest to the time-point of 28 days prior to the date of death was also extracted.

#### Greece

Due to the lack of a patient registry with meaningful completeness in Greece, we used hospital charts from two hospitals in different geographic areas to identify patients treated for BC from database inception (1/1/1992) until 31/12/2016. The individual patient charts were accessed and data were extracted regarding patient demographics (age at diagnosis, menopausal status), tumor pathologic characteristics (receptor expression, stage at initial diagnosis) and clinical information (date of primary disease diagnosis, date of diagnosis of metastatic disease, date and cause of death and all treatments received, including type of primary surgery, use of perioperative therapies and all lines for metastatic disease).

### Statistical analysis

Since this is a descriptive analysis of the use of futile chemotherapy in MBC, no formal statistical hypothesis was tested. Categorical variables were summarized in frequency tables. Continuous variables were summarized with descriptive statistics. Pearson’s Chi-squared test was used to compare categorical variables and Mann–Whitney U test for continuous ones. Overall survival was measured from the date of diagnosis of MBC until the date of death from any cause or the date of last follow-up. Overall survival was estimated using the Kaplan–Meier method and the comparisons were computed with the log-rank test. Logistic regression was performed to identify factors associated with the use of futile chemotherapy. An arbitrary level of 5% statistical significance (two-tailed) was used. IBM SPSS Statistics version 25 (IBM, NY, USA) was used to perform the statistical analyses.

## Results

### Patient characteristics and outcomes

#### Sweden

During the period 2010–2015, 1571 patients were diagnosed with metastatic BC in the Stockholm–Gotland region according to the population registry. Their clinical and demographic characteristics are presented in Table 1. At the time of data cut-off (26/3/2019), 202 patients (12.9%) were still alive, 1358 had died (86.4%) and 11 (0.7%) were lost to follow-up. After a median follow-up of 73.5 months (95% Confidence Interval [CI] 68.6–78.3), the median overall OS was 16.96 months (95% CI 15.4–18.4). The survival probability at 1, 3 and 5 years was 59.4%, 28.8% and 15.3%, respectively. Patients ≤ 60 years had longer OS compared to those older than 60 (24.96 months, 95% CI 20.6–29.2 versus 14.23 months, 95% CI 12.7–15.7, *p* < 0.001). In addition, ER-positive patients had improved OS compared to ER-negative ones (22.33 months, 95% CI 19.9–24.6 versus 7.66 months, 95% CI, 5.2–10.1; *p* < 0.001).

**Table 1:** Patients’ clinicopathologic characteristics

	*N* (%)	
	Swedish cohort	Greek cohort
Age		
Median (min–max)	68 (21–100)	57 (26–89)
≤ 60	458 (29.2)	575 (59.5)
> 60	1113 (70.8)	391 (40.5)
Menopausal status		
Premenopausal	428 (27.2)	348 (36.0)
Postmenopausal	1073 (68.3)	618 (64.0)
Histology	N/A	
Ductal		756 (78.3)
Lobular		73 (7.6)
Other		48 (4.9)
N/A		89 (9.2)
Estrogen receptor		
Positive	833 (53.0)	499 (51.7)
Negative	224 (14.3)	279 (28.9)
N/A	514 (32.7)	188 (19.5)
Progesterone receptor		
Positive	656 (41.8)	409 (42.3)
Negative	383 (24.4)	363 (37.6)
N/A	532 (33.9)	194 (20.1)
HER2		
Positive	66 (4.2)	215 (22.3)
Negative	406 (25.8)	485 (50.2)
N/A	1099 (70.0)	266 (27.5)

*HER2* human epidermal growth factor receptor 2, *N/A* not available

#### Greece

In total, 2501 BC patients were identified in hospital charts during the period 1992–2016. Of those, 966 patients developed metastatic disease. Their clinical and demographic characteristics are presented in Table 1. After a median follow-up of 110.9 months (95% CI 98.6–123.3), 459 patients were still alive (47.5%), 477 were dead (49.4%) and 30 had been lost to follow-up (3.1%). The median OS of patients in this cohort was 49.8 months (95% CI 45.6–54.1) and the corresponding 1-, 3- and 5-year survival probabilities were 87.6%, 62.4% and 42.7%, respectively. There was no difference in median OS according to patient age (*p* = 0.201) or hormone receptor status (*p* = 0.057).

### Descriptive analysis of the use of chemotherapy near the end-of-life

#### Sweden

At the time of data cut-off, 1358 patients had died; 59 (4.3%) of those didn’t have evaluable data regarding their treatment. During the last month of life, 315 patients (23.2%) received at least one chemotherapy dose. The types of administrated chemotherapy are shown in Table 2. Use of any chemotherapy regimen at this setting was more common among patients ≤ 60 years compared to > 60 years old (37.2% versus 19.3%, *p* < 0.001). There was no difference according to tumor ER status, since 23.2% of ER- and/or PR-positive versus 29.6% of ER- and PR-negative patients among those with known receptor status received any chemotherapy (*p* = 0.067). Moreover, a new chemotherapy regimen was initiated during the final 30 days of life in 114 patients (8.4%). Again, younger patients were more commonly started on a new regimen compared to those older than 60 (15.1% versus 5.9%, *p* < 0.001). In contrast, start of a new chemotherapy regimen was not affected by hormone receptor status in the primary tumor among patients with available receptor data (9.0% for ER- and/or PR-positive versus 10.4% for ER and PR negative, *p* = 0.576).

**Table 2:** Use of specific chemotherapy regimens at the final month of life in the Swedish cohort and per age group

Chemotherapy	All patients (%) (*n* = 315)	≤ 60 years (%) (*n* = 132)	> 60 years (%) (*n* = 183)
Capecitabine	78 (24.8)	32 (24.2)	46 (25.1)
Paclitaxel	46 (14.6)	19 (14.4)	26 (14.7)
Vinorelbine	31 (9.8)	10 (7.6)	21 (11.5)
Cyclophosphamide and methotrexate	28 (8.8)	10 (7.6)	18 (9.8)
Eribulin	33 (10.4)	18 (13.6)	15 (8.2)
Liposomal Doxorubicin	13 (4.1)	5 (3.8)	8 (4.4)
Epirubicin and cyclophosphamide	15 (4.7)	7 (5.3)	8 (4.4)
Platinum salts	5 (1.5)	3 (2.3)	2 (1.1)
Trastuzumab monotherapy	16 (5.0)	4 (3.0)	12 (6.6)
Other chemotherapy combinations	11 (3.5)	7 (5.3)	4 (2.2)
Chemotherapy and trastuzumab and/or lapatinib and/or pertuzumab combinations	28 (8.8)	11 (8.3)	18 (9.3)
Other	11 (3.5)	6 (4.5)	5 (2.7)

#### Greece

Out of the 477 patients with evaluable data that had died at the time of data cut-off, 222 patients received any chemotherapy regimen at the last month of life (46.5%). The median number of previous treatment lines in this group was four (range, 1–10). No significant difference in the receipt of chemotherapy near the end-of-life was observed according to age group (46.1% in ≤ 60 years versus 47.3% in > 60 years old, *p* = 0.081) or hormone receptor status in patients with available data (44.1% in ER- and/or PR-positive versus 53.7% in both receptors negative *p* = 0.594). The use of the various chemotherapy regimens is presented in Table 3. A new chemotherapy regimen was started during the final month of life in 93 patients (19.5%). Again, no difference was noted according to age (20.1% in ≤ 60 years versus 18.3% in > 60 years old, *p* = 0.839) or receptor status in the primary tumor (18.9% in ER- and/or PR-positive versus 21.0% in both receptors negative, *p* = 0.892).

**Table 3:** Use of specific chemotherapy regimens at the final month of life in the Greek cohort and per age group

Chemotherapy	All patients (%) (*n* = 222)	≤ 60 (%) (*n* = 142)	> 60 (%) (*n* = 80)
Capecitabine	7 (3.2)	5 (3.5)	2 (2.5)
Paclitaxel	12 (5.4)	4 (2.8)	8 (10.0)
Vinorelbine	5 (2.3)	3 (2.1)	2 (2.5)
Eribulin	2 (0.9)	1 (0.7)	1 (1.3)
Liposomal Doxorubicin	17 (7.7)	7 (4.9)	10 (12.5)
Platinum salts	3 (1.4)	3 (2.1)	–
Other drugs as monotherapy	19 (8.6)	15 (10.6)	4 (5.0)
Chemotherapy and Trastuzumab and/or Lapatinib and/or Pertuzumab combinations	21 (9.5)	14 (9.9)	7 (8.7)
Docetaxel-based combinations	40 (18.0)	26 (18.3)	14 (17.5)
Vinorelbine-based combinations	22 (9.9)	15 (10.6)	7 (8.7)
Chemotherapy and Bevacizumab combinations	23 (10.4)	16 (11.3)	7 (8.7)
Other chemotherapy combinations	51 (23.0)	33 (23.2)	18 (22.5)

### Predictors for the use of chemotherapy near the end-of-life

In univariate analysis, younger age (Odds Ratio [OR] 0.95; 95% CI 0.94–0.96; *p* < 0.001) and higher albumin levels (OR 1.04; 95% CI 1.01–1.07; *p* = 0.004) were significantly associated with the use of any chemotherapy regimen at the final month of life in the Swedish cohort, while ER status was not (OR 0.71; 95% CI 0.50–1.02; *p* = 0.068). Both factors remained significantly associated with the use of any chemotherapy in multivariable analysis (Table 4). In contrast, in multivariable analysis (Supplementary Table S1), age, receptor status and number of treatment lines were not associated with the likelihood of receiving chemotherapy at the final 30 days of life in the Greek cohort.

**Table 4:** Multivariable analysis of predictors for use of chemotherapy in the last 30 days of life in the Swedish cohort

Variable	Odds ratio	95% Confidence interval	*p*
Age	0.95	0.94–0.96	< 0.001
Albumin	1.05	1.02–1.09	0.002
ER status	0.83	0.54–1.27	0.401

*ER* estrogen receptor

## Discussion

Data on treatment of MBC near the end-of-life are scarce, making patient management more intuition-based rather than data-driven. The use of “futile” chemotherapy may lead to impaired quality of life for patients in the last stage of MBC, underscoring the need to recognize how widespread this phenomenon is in an effort to eventually mitigate it. In this retrospective cohort study, we describe the real-world use of chemotherapy near the end-of-life in areas of Sweden and Greece. The Swedish cohort was identified from a population-based registry with adequate completeness, providing a representative sample of the MBC population. Data on the Greek cohort were extracted from hospital charts, which represents a selected population fit enough to receive active cancer treatment. Consequently, results from the two cohorts are not comparable with each other, especially considering that approximately a fifth of the Swedish patients never received any active oncologic treatment but were nevertheless included in the registry.

The median OS in the Swedish cohort was 16.96 months, which represents a marginal improvement when compared to the results from previous decades in the same geographic area [8]. This slow improvement reflects on the one hand the therapeutic advances, but also the increasing use of adjuvant polychemotherapy and endocrine therapy, which in turn greatly decreases the number of metastatic relapses but enriches them with treatment-resistant patients [12]. However, OS was clearly worse as compared to results reported in modern clinical trials, corresponding to the fact that participants in population-based studies are generally less fit, older and with more comorbidities than that of clinical trials [13], but also that many of the patients included in the survival analysis did not receive any treatment. In contrast, the median OS in the Greek cohort was in line with outcomes reported from contemporary trials, which is likely attributed to the selected population which was considered fit enough to receive anticancer treatment.

During the last month of life, 23.2% of patients in the Swedish cohort received at least one chemotherapy treatment. Expectedly, younger patients and those in better condition—using albumin as a surrogate [11]—were more often treated during this late phase of the disease. This is consistent with observations regarding tolerance of chemotherapy among elderly patients due to age-related developments in human physiology including decline in liver and kidney function and marrow reserves, which affects clinical decision-making [14–16]. Other factors which may have influenced this under-representation of elderly patients, such as refusal of anticancer treatment—which is far more common among older and more ill patients [17] and delays in seeking medical assistance, in referral to specialized oncology care or in treatment initiation cannot of course be excluded. An even higher usage of chemotherapy in general and introduction of a new regimen in particular was noted in the Greek cohort. These rates are considerably higher compared to a previous study by Wallington et al. where 30-day mortality following the last administration of chemotherapy in patients with MBC was 7% [18]. Although cross-trial comparisons can be misleading since differences in methodologies, data sources and study endpoints may account for the observed variations in chemotherapy use, cross-cultural differences in both physician and patient perceptions and expectations concerning the disease, treatment and prognosis possibly affect patient management. In addition, the choice of chemotherapeutics used during the last of month of life exhibited significant variation, a fact that is consistent with, and caused by, the lack of evidence-based recommendations regarding heavily pretreated patients. In the Swedish cohort, chemotherapy was more commonly given as monotherapy administered orally. This may be the preferable choice for late-stage patients wanting to avoid regular contacts with the hospital for intravenous therapy. Such a preference was not noted in the Greek cohort though, further highlighting the different approaches employed.

Previous studies have reported on the frequency of aggressive interventions, including chemotherapy administration, in patients with advanced malignancies with varying results. Although two studies from Canada showed that only 6.3% and 22.4% of patients received chemotherapy or experienced aggressive medical interventions (such as emergency department visits or intensive care admissions) respectively, both endpoints were observed more frequently among patients with MBC [19, 20]. The latter was confirmed by another study reporting high rates of aggressive end-of-life care among patients with MBC [21]. Interestingly, chemotherapy use at the last month of life in patients with multiple tumor types was reported in a fourth of all patients in two studies from Sweden and Italy, which is similar to the Swedish cohort in the present study [22, 23], although the number of patients with MBC was too low to allow for meaningful comparisons.

Following the surveying of chemotherapy use at the end-of-life, the next step is efforts aiming to limit it, considering the clear potential benefit: overtreating patients with palliative chemotherapy does not improve survival [24], exposes patients to unnecessary toxicity, negatively impacts their quality of life and has been shown to be associated with aggressive interventions near the end-of-life [25]. Importantly, mutually reinforcing attitudes by both patients and physicians may further aggravate this phenomenon, indicating that physician-centered interventions might not adequately remedy this phenomenon [26]. As a result, mitigating efforts should also focus on patient education, frank communication and frequent discussions on treatment goals, the disease course and eventual prognosis, although the latter is notoriously hard to predict [27].

Besides selection bias, inherent in retrospective studies, several weaknesses of this study should be acknowledged. Considerable data missingness and differences in collected variables between the two cohorts do not permit any meaningful comparisons of possible factors that affect the decision to continue with chemotherapy at this stage of the disease. Furthermore, data regarding the cause of death were not collected. Both toxic death due to palliative chemotherapy for MBC, although a relatively rare event [18], and competing non-cancer related causes of death may have affected the results so that the true rates of “futile” chemotherapy administration may be lower than those presented. Finally, factors such as receptor status at the metastatic setting which has been shown to differ compared to the primary tumor [28], socioeconomic status, healthcare setting, treating physician’s experience and others that could affect chemotherapy use were not evaluated.

## Conclusion

In conclusion, use of chemotherapy near the end-of-life was fairly common, especially among younger patients, while a wide variety of agents was shown to be used in this setting. Prospective assessment of mitigation strategies is warranted.

## Electronic supplementary material

Below is the link to the electronic supplementary material.

Supplementary file1 (DOCX 12 kb)
