# Supplementary material for: Chemotherapy use near the end-of-life in patients with metastatic breast cancer
Source: Breast Cancer Res Treat. 2020 May 7;181(3):645–51. doi: 10.1007/s10549-020-05663-w (PMC7220858; doi:10.1007/s10549-020-05663-w)
Supplement: Supplementary file 1 — Supplementary file1 (DOCX 12 kb) [file 10549_2020_5663_MOESM1_ESM.docx]

**Supplementary Material to**

**Chemotherapy use near the end of life in patients with metastatic breast cancer**

Luisa Edman Kessler, Johnny Sigfridsson, Dora Hatzidaki, Jonas Bergh, Theodoros Foukakis, Vasilios Georgoulias, Alexios Matikas

**Supplementary Table S1.** Multivariable analysis of predictors for use of chemotherapy in the last 30 days of life in the Greek cohort.

| **Variable** | **Odds Ratio** | **95% Confidence Interval** | **p** |
| --- | --- | --- | --- |
|  |  |  |  |
| Age | 1.3 | 0.8 – 2.1 | 0.334 |
| Chemotherapy Lines | 1.0 | 0.6 – 1.7 | 0.922 |
| Hormone receptor status | 1.2 | 0.7 – 1.9 | 0.492 |
